# Evaluation of Radiological Health Risks in Popularly Consumed Brands of Sachet Water in Nigeria

**DOI:** 10.3389/fpubh.2022.917422

**Published:** 2022-07-18

**Authors:** Kehinde Aladeniyi, Christopher Jimoh Olowookere, Mayeen Uddin Khandaker, Sultan J. Alsufyani

**Affiliations:** ^1^Department of Physics, Federal University of Technology, Akure, Nigeria; ^2^Department of Physics, University of Medical Sciences, Ondo, Nigeria; ^3^Centre for Applied Physics and Radiation Technologies, School of Engineering and Technology, Sunway University, Bandar Sunway, Malaysia; ^4^Department of General Educational Development, Faculty of Science and Information Technology, Daffodil International University, Dhaka, Bangladesh; ^5^Department of Physics, College of Science, Taif University, Taif, Saudi Arabia

**Keywords:** sachet drinking water, gamma-ray spectrometry, radium and thorium contents, radiation health risks, activity concentrations

## Abstract

Radiological investigation of 35 brands of most popularly used sachet drinking water in Ondo state, Nigeria has been carried out using a spectrometric method for evaluating the concomitant health risks to the members of the public. Activity concentrations of the investigated radionuclides ^40^K, ^226^R, and ^228^Ra were in the range from 16.35 ± 4.10 to 199.94 ± 38.40 Bq L^−1^ with an arithmetic mean (AM) of 66.22 ± 54.99 Bq L^−1^, from 1.35± 0.79 to 17.06 ± 5.13 Bq L^−1^ with an AM of 6.88 ± 3.66 Bq L^−1^, and from 1.95 ± 0.08 to 17.22 ± 3.87 Bq L^−1^ with an AM of 9.49 ± 4.98 Bq L^−1^, respectively. The determined annual effective doses and the corresponding excess lifetime cancer risks due to ^226^Ra and ^228^Ra were found to exceed the acceptable limits of 0.1 mSv y^−1^ and 10^−3^ respectively, as suggested by the World Health Organization (WHO). This implies a non-negligible carcinogenic health hazard due to the intake of the surveyed drinking water, especially for the lactating babies (0–1) y and teenagers (12–17) y. The data from this research may form an invaluable component of radiometric values of the database in Nigeria, as well as the world for setting up guidelines and control policies for the use of sachet water.

## Introduction

Water accounts for about 70% of the human body weight, therefore it is indispensable for life. Moreover, it is a vital compound for industrial, agricultural, commercial, and domestic applications. In this regard, the accessibility and quality of water for human use are very important. As a natural solvent, water not only dissolves and stores almost all substances it comes in contact with but also serves as a means of transporting the substances from one point to another even within the human body (1–3). The substances are observed to vary in quantity and their levels of toxicity. Among the substances contained in varying amounts in water bodies, a trace amount of radionuclides is also found to originate from both natural and artificial sources. Radionuclide distribution in the water body is heterogeneous and dependent greatly on the local geology from which the water is sourced (1, 4–7). Therefore, the local geology dictates a good degree of the level of radiation to which human beings are exposed unavoidably. Domestic use of radiologically contaminated water may become a source of both external and internal radiation exposures to human bodies. This may in turn lead to unwanted health hazards (8). Carcinogenic effects on the lung, kidneys, bladder, stomach, and disease conditions such as mutagenicity, leukemia, etc., are possible health effects of overexposure to water-containing radionuclides when ingested.

The major sources of water in Nigeria are streams, rivers, boreholes, and drilled wells out of which commercial products such as bottled and sachet water are produced (9). Notably, in recent times, the proliferation of sachet water, popularly referred to as “Pure water,” shows unprecedentedly high demand in Nigeria. The demand might have resulted from the difficult access to pipe-born water, perceived level of purity, and low prices of the product among others. Although pure water is sourced from the earth's crust (drilled wells/boreholes) prior to a series of water purification processes, the end products (sachet water) are likely to retain their radioactive contents. This may be because no specific procedure has been developed/dedicated for their removal by most of the water companies in Nigeria. At elevated levels, the natural radionuclide that constitutes a global concern in respect of human health hazards is radium. It is highly soluble in water and gets dissolved easily in the waterbody when the surrounding underground rocks and soil are bathed. As a result, through the ingestion of drinking water, a significant amount (~20%) of radium gets absorbed into the bloodstream which may, in turn, pose possible detriments to several organs of the body. For instance, different sarcoma and carcinoma have been linked to the presence of radium in human bodies. The two isotopes of radium, that is, ^228^Ra and ^226^Ra, the progeny of ^232^Th and ^238^U decay series respectively, are the principal radiotoxic elements in the waterbody as they release alpha- and beta-particles upon decays (10). The knowledge of the concentrations of these radioisotopes in water bodies is essential for the evaluation of radiation hazards or exposure to the population. To avoid the overexposure to hazardous radium *via* consumption of drinking water, many studies on radioactivity levels in water have been conducted in different parts of the world including Nigeria (11–20). The results from such studies were compared with the international reference levels assigned for various radionuclides by regulatory bodies like the World Health Organization (WHO) and European Commission (EU). In Nigeria, Avwiri et al. (21) studied the concentrations of natural radionuclides ^238^U, ^232^Th, and ^40^K in water samples fetched from boreholes in 29 locations at Portacourt, Cross-River state. Even though the mean activity concentrations of the radionuclides were found to be relatively high, the corresponding evaluated mean annual effective doses did not exceed the recommended limits. This means that the sampled borehole water may not pose any radiological hazards to the end-user. This result is in clear contrast with the results reported for drinking water by Ajayi and Owolabi (22) in Akure, Ondo state (dug well water), Ajayi and Achuka (23) in Ogun state (drilled and dug well water), Ajayi and Adesida (15) in Akure, Ondo state (few sachets produced water), Aladeniyi and Aladenika (19) in Owo, Ondo state (Sachet-packaged water), Ayodele et al. (23) in nine cities of both Ondo and Ekiti states (dug well water) and Ayodele et al. (24) in some cities also in both Ondo and Ekiti states (drilled well water). In all of these studies, both the drilled and dug well waters were recommended for urgent treatments, and the sampled population was advised to take less of the sachet water/sachet-packaged water to avoid overexposure to unnecessary radiation. It is reasonable to assume that the concentrations of radionuclides in either bottled water or sachet packaged water produced and consumed in any location are functions of radionuclide contents of the unprocessed water from which the products are sourced, the methods of treatment, and the water resources.

Generally, in Nigeria, bottled or sachet packaged waters have become the major source of drinking water. This may be due to awareness of very many deadly diseases that are associated with polluted water and the unavailability of pipe-borne water. This is evident from the influx of brands of sachet packaged waters flooding marketplaces and shops in most areas of Nigeria, Ondo state inclusive (15). It is, therefore, necessary to ensure that the quality of products is safe for human consumption.

This study is centered on the evaluation of ^226^Ra (^238^U), ^228^Ra (^232^Th), and ^40^K concentrations in the sachet water consumed in Ondo state, Nigeria with a view to investigating the associated radiological risks. The obtained data are not only hoped to boost the quantity and quality of the existing database but also will help to ensure the sale of safe sachet water, reducing radiological risks to the consumers.

## Materials and Methods

### Study Area

**Figure 1:**
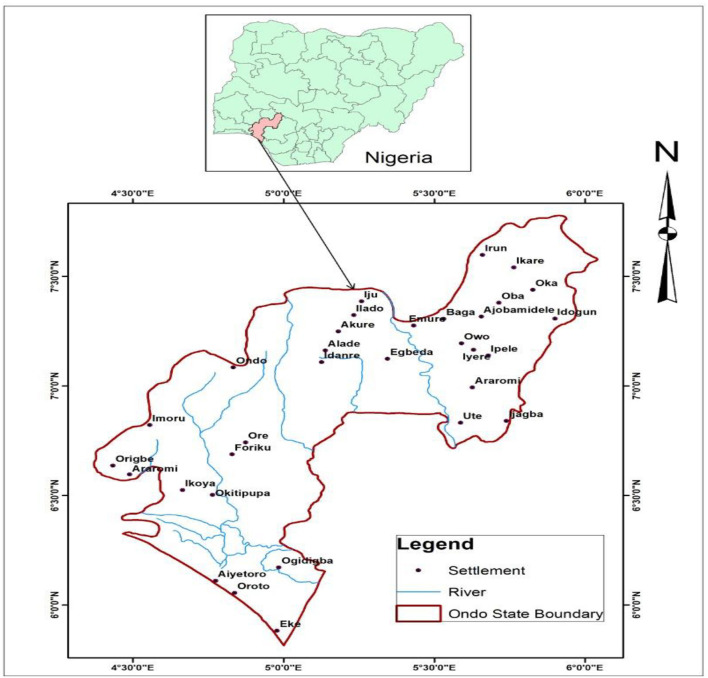
Map of the study area.

The study area is the entire Ondo state (Figure 1), an oil-producing state and one of the six states in the South-Western region of Nigeria. It can be found within latitudes 5° 45' and 7° 52'N and longitude 4° 20' and 6° 05'E with a land area of about 15,500 km^2^. The area had a recorded population of 3,460,877, results shown from the most recent national population census conducted in 2006 (25, 26). Concerning its topography, it comprises lowland and hills with two prominent hills (250 m above sea level) found in the Idanre and Akoko areas of the state. It shares boundaries in the North with Edo state, in the West with Osun and Ogun states, and in the East with Ekiti and Kogi States (27). The state is underlain with the basement complex/rocks and sedimentary rocks, integrated pre-Cambrian rocks units, comprising granites, pegmatites, migmatite, gneisses, schist/meta-diments, and quartzite. The state is located in the tropical rain forest region and composed of two seasons, namely, dry season (November–March) and wet season (April-October) with a relative humidity in the range of 70–80% recorded yearly, and the annual temperature ranges from about 18 ° C on a very cold day and 34 ° C on a very hot day (28).

### Sample Collections and Preparation

Thirty-five samples from 35 brands of popularly consumed sachet-packaged drinking water (sachet water) were purchased and measured for natural radioactivity levels. The brands were representatives of a total of 35 sachet-packaged water-producing companies/factories located in different geological sites across the Ondo state in Nigeria. All the brands have been registered by the National Agency for Food and Drug Administration and Control (NAFDAC) as indicated by the labels placed on the water sachets. The sachets used by the 35 factories are thermoplastic bags (polyethylene terephthalate) of approximately 0.5 liters and heat-sealed at one end. Two sachets of water from each brand were emptied into 1-l keg (container) and acidified immediately with 11 M HCl at a rate of 10 ml to prevent the absorption of radionuclides in the samples by the container wall (23). The sampled brands were anonymously labeled as Awa1, Aw2, Aw3, and Aw35. The prepared water samples were transported to the Center for Energy Research and Development (CERD) laboratory in Obafemi Awolowo University, Ile-Ife Osun state Nigeria. Thirty-five radon impermeable Marinelli beakers were washed with acidified water, dried, and filled with the prepared water samples from the kegs (one for each). The beakers were sealed and kept for at least 30 days prior to the spectrometric analysis to allow for secular equilibrium between the radium isotopes and their daughter radionuclides (8, 29). The Marinelli beakers have the same geometry as the standard sample container used in this study. The standard sample is a multi-radioisotope gamma source (^137^Cs, ^60^Co, ^207^Bi) with activity homogeneously distributed.

### Sample Measurements

A gamma ray spectrometric technique was used to measure the activity concentrations of the samples. The system comprises a 7.6 × 7.6 cm NaI (Tl) detector (Bicron Corp model 3M/3), shielded from background radiation by a cylindrical lead shield of thickness 5.5 cm and kept in the CERD's laboratory. The detector was coupled to a set of electronic systems: a pre-amplifier, an amplifier (Canberra Model 2022), and an Analog-to-digital converter (Canberra Model 8075), which sends output signals to a Canberra S100 Multi-Channel-Analyzer (MCA). The detector energy calibration and efficiency evaluation were performed using a standard water sample of IAEA (MBSS 197-92-16-1010, No: 09-92). The concentrations of the radionuclides ^226^Ra (^238^U series), ^228^Ra (^232^Th series), and the non-series radionuclide ^40^K were respectively measured through the photo peaks of 1,764 keV (Iγ = 15.30%) emitted from short-lived nuclide ^214^Bi, 2,614 keV (Iγ = 99.754%) emitted from nuclide ^228^Ac, and 1,460 keV (Iγ = 10.66%) for ^40^K. Each of the prepared samples was counted for 25,200 s. The effect of possible background radiation was eliminated. In this case, an empty container having the same geometry as the sample-filled Marinelli beakers and the standard-filled container was counted. The counts were subtracted from counts under the corresponding photopeaks of interest (19).

### Activity Concentration of the Radionuclides

In the sampled water, activity concentrations of the radionuclides were obtained using the comparative method as presented in equation (1) (19, 29, 30);


(1) $$\begin{array}{r} \frac{A_{SR}}{A_{SD}}=\frac{N_{nS}}{N_{nD}} \end{array}$$

where A_SR_ and A_SD_, are the activity concentrations in Bq L^−1^ of the sample and the standard sample with regard to radionuclide R, respectively. N_nS_ and N_nD_ are the net count rates under the region of interest for the sample and the standard in respect of the radionuclide, respectively. The minimum detectable activity (MDA) was determined using the Equation (2) as reported in (31):


(2) $$\begin{array}{r} MDA=\frac{2.71+\left(4.66\;\times \;\sigma \right)\;}{\varepsilon_{\gamma }\times \;t\;\times \;I_{\gamma }\times m} \end{array}$$

where σ is the standard deviation in the absence of any isotope during measurement (i.e., only the background and interference terms are present), ε_γ_ is the efficiency of the detector at respective gamma-ray energy, t is the counting time, *I*_γ_ is the branching ratio or the intensity of γ-ray, and m is the mass of the sample. The MDA values for the radionuclides of interest in this study were found to be 1.30 Bql^−1^ for ^226^Ra, 1.82 Bql^−1^ for ^228^Ra, and 7.65 Bql^−1^ for ^40^K. All relevant radiological indices were computed from the determined activity concentrations of the radionuclides in the samples.

### Computation of Annual Effective Dose and Carcinogenic Risks

The presence of radionuclides in ingested water is a matter of major health concern as internal exposure to ionizing radiation may occur within the body. The exposure may lead to radiological acute and chronic health effects depending on the concentration of the decaying radionuclides. To control the unexpected radiation exposures with respect to water consumption, a need for estimating individual annual effective dose (AED) is required. This can be achieved by applying equation (3) (11, 19, 32);


(3) $$\begin{array}{r} AED=A_{R}\times CR\times DC \end{array}$$

where A_R_ (Bq L^−1^) stands for activity concentration of the radionuclides of interest, CR is the annual consumption rate of water in (L y^−1^), and DC is the activity-to-dose conversion factor (Sv/Bq), obtained from the IAEA's document (33) for radionuclides of interest (^226^Ra, ^228^Ra, and ^40^K). The annual effective dose does not only depend on the ages of the exposed individuals but also depends on the annual consumption rates of the water. Six age groups, that is, <1 y, 1-2 y, 2-7 y, 7-12 y, 12-17 y, and >17 y with their corresponding rates of water consumption 250, 300, 350, 400, 550, and 730 (L/y), respectively (15, 34, 35), were used for estimating the AED. In a similar manner, radiological risks/excess life-time carcinogenic risk (ELCR), that is, morbidity and mortality cancer risks were evaluated for radium isotopes (^226^Ra and ^228^Ra) using equation (3) (36, 37).


(4) $$\begin{array}{l} \begin{array}{ccc} ELCR & = & A_{R}(BqL^{-1})\;\times \;MC\;(Bq^{-1})\times 2\;(Ld^{-1}) \end{array}\\ \begin{array}{cc} \;\times & 365\;(dy^{-1})\;\times \;70\;(y) \end{array} \end{array}$$

where, the term A_R_ (BqL^−1^), MC (Bq^−1^), 2 (Ld^−1^), 365 (dy^−1^), and 70 y are the activity concentration of each radionuclide of interest in the samples, mortality/morbidity risk coefficients, daily intake of drinking water, days/year, and duration of life, respectively. The mortality/morbidity risk coefficients for ^226^Ra and ^228^Ra are 7.7 × 10^−9^/1.0 × 10^−8^ Bq^−1^ and 2.00 × 10^−8^/2.88 × 10^−8^ Bq^−1^, respectively (36).

### Statistical Consideration

Both descriptive statistics (arithmetic means with standard deviation) and inferential statistics (independent two-sample *t*-test and Wilcoxon rank sign tests) were applied to the measured activity concentrations of radionuclides in the study using IBM SPSS (Version 25) software with 0.05 α-level of significance.

## Results and Discussion

Table 1 shows the activity concentrations in (Bq L^−1^) for the investigated radionuclides (^40^K, ^226^Ra, and ^228^Ra) in the sachet-packaged water samples. The activities ranged from 16.35 ± 4.10 (Awa21) to 199.94 ± 38.40 Bq L^−1^ (Awa28) with an arithmetic mean (AM) of 66.22 ± 54.99 Bq L^−1^, from 1.35± 0.79 (Awa31) to 17.06 ± 5.13 Bq L^−1^ (Awa5) with an AM of 6.88 ± 3.66 Bq L^−1^, from 1.95 ± 0.08(Awa30) to 17.22 ± 3.87 Bq L^−1^ (Awa7) with an AM of 9.49 ± 4.98 Bq L^−1^ for ^40^K, ^226^Ra, ^228^Ra, respectively. The activity concentrations of ^40^K in samples Awa7, Awa25, and Awa34, ^226^Ra in samples Awa6, Awa17, Awa21, Awa24, Awa30, and Awa32, and ^228^Ra in samples Awa19, Awa22, Awa26, Awa28, and Awa34 were not detected or are below the MDAs. The geochemistry of the parent radionuclides (for radium isotopes), the geological condition of the origin of water sources, and the depth of the rocks hosting the aquifers for the water sources may be responsible for the observed distribution of radionuclides in the samples. Also, the interaction between the water and the solid phases over long periods in the deep water table may be the other reason for the variations in the concentrations of the surveyed radionuclides in the investigated water samples. Apart from the non-detectable concentrations of ^40^K in the Awa7, Awa25, and Awa34 samples, the concentration of the radionuclide is higher in each of the samples than the corresponding concentrations of the radionuclides ^226^Ra and ^228^Ra. This trend has always been observed generally in various studies conducted for the radioactivity in surface soil samples (38, 39), in building materials (40–42), and in drilled and dug well waters (22, 24, 43, 44), all from the region to which the study area belong. As a primordial isotope with a half-life of 1.28 × 10^9^ years, ^40^K occurs in abundance in all terrestrial media and its activity depends greatly on the local geology in any location like other radionuclides. Although ^40^K is a source of radiation that may enter human bodies through ingestion of contaminated water and food, it is considered an essential element as well as it is homeostatically controlled by the body systems upon ingestion. Therefore, the establishment of a guideline value for ^40^K for its control is not very necessary because its concentration in drinking water is not likely to rise to a level of health concern (45). The average value, 66.22 Bq L^−1^ evaluated for ^40^K is extremely higher than 0.194, 0.105, and 0.229 Bq L^−1^ reported from Jordan in tap water, drilled well-containing tap water, and rainwater, respectively (46). Also, the value exceeds the reported value of 14.16 Bq L^−1^ from Yemen in drinking surface water samples (18); 0.103 Bq L^−1^ reported from Turkey in surface and tap water (13); and 0.141, 2.19, 47.52, and 0.688 Bq L^−1^ in bottled water reported from Pakistan, Turkey, Iran, and Egypt, respectively (37, 47–49). In the case of the radium isotopes (^226^Ra and ^228^Ra), which have attracted global health concern at high levels in water and food substances, a close observation in Table 1 shows that the concentrations of ^228^Ra in 57% (20 samples) of the investigated samples are higher than the corresponding concentrations of ^226^Ra. This indicates that the concentrations of thorium in the aquifers from which the water samples were sourced are greater than the concentrations of the uranium. This trend agrees with the reports of studies conducted for activity and concentrations of ^226^Ra and ^228^Ra in ground waters from Saudi Arabia, China, Brazil (14, 50–53) and in drinking water sampled from private wells in Nigeria (22). To fix the unbalanced research design which occurred due to some values of activity concentrations of the investigated radium isotopes (^226^Ra and ^228^Ra) that fell below the MDAs, all the 24 pairs of activity concentration values with no NDs from Table 1 were selected. This is to have a meaningful statistical comparison between the two groups of the isotopes. Adopting an α-level of 0.05 (level of significance), Shapiro–Wilk's tests for normality distributions of the obtained data were carried out. The Shapiro–Wilk's tests indicates a normal distribution of data for the two groups, W_R−226_ (24) = 0.95, *P* = 0.31 for ^226^Ra and W_R−228_ (24) = 0.92, *P* = 0.07 for ^228^Ra. An independent *t*-test was conducted, taking the concentrations of the radionuclides (^226^Ra and ^228^Ra) of interest as the dependent variable and the isotopes of the radium being the two levels of the independent variables (radium), indicating that there is a statistically significant difference between the activity concentrations for ^226^Ra (M = 7.3, SD = 3.7) and ^228^Ra (M = 10.1, SD = 4.8); t_(46)_ = −2.23, *P* = 0.031. This implies that the two groups are different from each other in terms of the concentrations of the surveyed radionuclides. The observed higher content of ^228^Ra in the majority of the investigated samples as compared to the content of ^226^Ra may be attributed to the geology of the water sources, geochemistry of the parent radionuclides, and the interaction of water with the surrounding soil and rocks in the aquifers where various unprocessed waters were sourced for the production of the sachet water brands (11, 50, 54). Moreover, it can be observed that the concentrations of ^226^Ra and ^228^Ra in each of the surveyed brands of sachet water exceeded the recommended limits (1 Bq L^−1^ for ^226^Ra and 0.1 Bq L^−1^ for ^228^Ra) given by WHO (45). This implies that the water factories of the brands have not employed any means of eliminating/reducing the radionuclide contents in the water during production or have not regularly monitored the end products (sachet water) against radionuclide loads for quality control purposes. The results are in agreement with the reports of Ajayi and Adesida (15), Aladeniyi and Aladenika (19) for radiological studies carried out on different brands of sachet water in Nigeria. Although none of the total 10 brands of the sachet water studied by Aladeniyi and Aladenika (19) was present in this study, 7 brands (Awa2, Awa7, Awa15, Awa19, Awa20, Awa22, and Awa31) of the total brands (15) of sachet waters investigated by Ajayi and Adesida (15) were by chance included in this study. Using the data from the seven brand samples and the corresponding samples in this study, the two sets of data were subjected to a non-parametric test (Wilcoxon rank sign tests). This is an alternative test to paired sample t-test to ascertain if there are significant changes in the concentrations of the surveyed radionuclides as a result of possible water treatments suggested to the sachet-water producers by Ajayi and Adesida (15) for removing radionuclides in the water samples. The test revealed that no significant changes were observed in the concentrations of the surveyed radionuclides (z = −1.690, *P* = 0.091) for ^40^K, (z = −0.676, *P* = 0.499) for ^226^Ra, and (z = −1.352, *P* = 0.176) for ^228^Ra, indicating that no water treatments for the removal of radionuclides have been applied to the sachet water production processes by the producers against the remedial suggestion by Ajayi & Adesida in 2009. Table 2 shows the comparison of the ranges of surveyed radionuclide concentrations of this study with the reported values of similar studies around the world. It can be observed that all the water samples of various sources investigated in Nigeria contain relatively higher range of activity concentrations of the naturally occurring radionuclides. Consequently, this calls for serious health concerns and the creation of a standing policy to ensure quality control of the production of potable water in Nigeria.

**Table 1:** Activity concentrations of the radionuclides in the samples.

**Sample codes**	**Activity concentration (Bq L** ^**−1**^ **)**		
	**^**40**^K**	**^**226**^Ra**	**^**228**^Ra**
Awa 1	154.22 ± 21.08	13.02 ± 3.25	13.19 ± 3.04
Awa 2	138.73 ± 27.54	4.65 ± 2.02	15.41 ± 5.31
Awa 3	21.79 ± 5.76	13.27 ± 3.01	12.76 ± 2.08
Awa 4	76.64 ± 21.67	3.35 ± 0.98	14.60 ± 4.08
Awa 5	85.37 ± 18.09	17.06 ± 5.13	14.63 ± 3.31
Awa 6	28.57 ± 9.03	ND	14.49 ± 4.31
Awa 7	ND	10.95 ± 3.32	17.22 ± 3.87
Awa 8	96.84 ± 16.76	8.64 ± 2.12	15.95 ± 4.88
Awa 9	44.36 ± 13.78	5.24 ± 1.91	9.16 ± 2.99
Awa 10	67.47 ± 18.34	8.73 ± 1.47	6.34 ± 2.39
Awa 11	48.15 ± 11.97	6.38 ± 2.12	16.62 ± 4.99
Awa 12	92.08 ± 19.65	3.14 ± 1.22	14.35 ± 5.01
Awa 13	117.92 ± 44.97	6.87 ± 2.08	8.19 ± 3.63
Awa 14	198.62 ± 59.65	10.48 ± 3.42	7.92 ± 2.22
Awa 15	137.58 ± 34.21	9.88 ± 2.32	14.97 ± 6.09
Awa 16	140.14 ± 21.54	5.39 ± 1.65	12.23 ± 4.31
Awa 17	41.10 ± 12.99	ND	14.73 ± 3.55
Awa 18	25.22 ± 11.80	7.44 ± 2.32	4.62 ± 1.30
Awa 19	19.86 ± 5.41	5.17 ± 1.62	ND
Awa 20	36.16 ± 11.37	8.38 ± 2.31	7.26 ± 2.15
Awa 21	16.35 ± 4.10	ND	2.46 ± 1.12
Awa 22	41.98 ± 13.45	3.60 ± 1.02	ND
Awa 23	104.60 ± 15.87	3.07 ± 1.01	2.67 ± 1.08
Awa 24	26.46 ± 8.21	ND	4.26 ± 1.20
Awa 25	ND	5.47 ± 2.18	8.43 ± 2.27
Awa 26	31.25 ± 7.06	9.55 ± 2.13	ND
Awa 27	18.53 ± 8.11	3.18 ± 1.51	2.63 ±0.63
Awa 28	199.94 ± 38.40	2.23 ± 0.48	ND
Awa 29	16.60 ± 5.78	6.65 ± 2.12	8.20 ± 1.87
Awa 30	17.06 ± 5.21	ND	1.95 ± 0.08
Awa 31	18.75 ± 7.19	1.53± 0.79	5.24 ± 1.43
Awa 32	19.68 ± 6.67	ND	4.62 ± 1.87
Awa 33	17.74 ± 8.43	8.62 ± 2.14	6.34 ± 2.39
Awa 34	ND	3.63 ± 1.62	ND
Awa 35	19.20 ± 5.56	4.02 ± 2.01	3.15 ± 1.41

*ND, Not detected*.

**Table 2:** Comparison of ^40^K, ^226^Ra, and ^228^Ra concentration ranges in various water types with results reported from other countries.

**Countries/Reference**	**Water types**	**Activity concentrations (mBq L** ^**−1**^ **)**		
		**^**40**^K**	**^**226**^Ra**	**^**232**^Th/^**228**^Ra**
Bangladesh (8)	Bottled Drinking water	–	31.1 ± 7.2–86.4 ± 4.8	22.6 ± 3.8–71.0 ± 14.2
Malaysia (11)	Bottled mineral water	21.12 ± 1.74–25.31 ± 1.84	1.45± 0.28–3.30± 0.43	0.65 ± 0.18–3.39 ± 0.38
Jordan (46)	Tap water	101–342	<19–302	24–119
Turkey (13)	Surface and tap waters	118–91.1	13.7–10.8	3.6–2.1
Yemen (18)	Drinking groundwater	7.84 ± 0.25–18.02 ± 0.57	0.86 ± 0.067–3.09 ± 0.12	0.46 ± 0.02– 2.01 ± 0.07
Iran (48)	Bottled mineral water	<1.29 to 389.17	<0.03 to 3.88	<0.013–13.75
Serbia (16)	Drinking water	–	0.23–7.8	<0.02–0.87
S. Arabia (14)	Groundwater		12 ± 10–590 ± 49	62 ± 70–2120 ± 80
Croatia (17)	Bottled drinking water		36.7–52.1	11.6–35.8
Turkey (12)	Drinking water samples	<47–2880	<27–2431	<36–270
Pakistan (47)	Bottled drinking water	92 ± 5–216 ± 10	8 ± 0.6–15 ± 2	4 ± 0.5–6 ± 0.8
Egypt (55)	Groundwater	25–344	8–40	3–19
Finland (56)	Drilled well water	–	<10–1000	30–300
Nigeria (22)	Dug well water	350–29010	570–26860	200–60060
Nigeria (15)	Sachet Drinking water	570–34080	2220–15500	40 −7040
Nigeria (23)	Dug well water	1740–4690	2890–7790	
Nigeria (19)	Sachet packaged water	21000–142000	600–11600	4500–18600
Nigeria (43)	Dug well water	35810–70380	6430–12590	1590–3750
Nigeria (24)	Drilled well water	45420 −467610	7080–56680	2250–35610
Nigeria (This study, 2021)	Sachet drinking water	16350–199940	1530–17060	1950–17220

**Figure 2:**
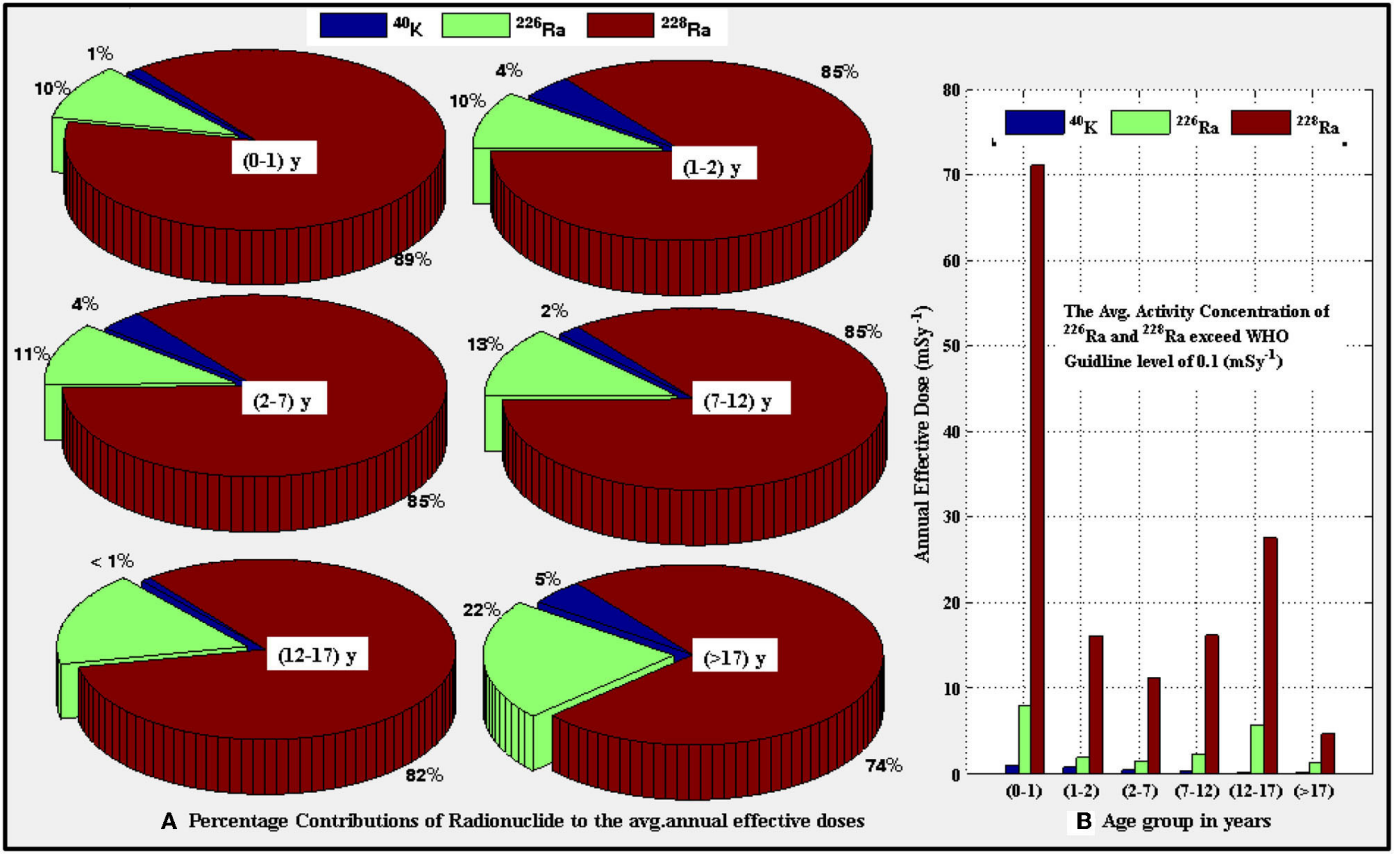
**(A)** Percentage contributions of the radionuclides to the annual effective dose for different age range **(B)** Distribution of the mean annual effective dose due to the presence of ^40^K, ^226^Ra, and ^228^Ra for various age range.

Table 3 shows the descriptive statistics of the annual effective doses (AED) received in mSvy^−1^ following the consumption of the surveyed radionuclides *via* drinking. The values are dependent on the age groups and radionuclide types. Based on the average values, it can be observed that the values of AFD for ^40^K, ^226^Ra, and ^228^Ra changed from 0.28 (12–17 y) to 1.03 (0–1 y) mSv y^−1^, from 1.41 (>17 y) to 8.09 (0–1y) mSvy^−1^, and 4.78 (>17 y) to 71.15 (0–1 y) mSvy^−1^, respectively. The percentage contributions of the investigated radionuclides to the total AED calculated for different age groups are presented in Figure 2A. In each of the age groups, ^228^Ra made the highest contributions, followed by the contribution from ^226^Ra, and the contribution of ^40^K was the least. This may be a reflection of the geological features of the rocks hosting the aquifers from which the raw water for sachet water production was sourced and solubility differences among the radionuclides. The values of AED for ^40^K were only included for the purpose of comparisons. The annual effective doses for all age groups due to ^226^Ra and ^228^Ra are higher than the recommended limit of (0.1 mSv^−1^) suggested by the World Health Organization (45). Although the doses for all the age groups exceeded the permissible limit, the most vulnerable group to internal radiation exposure due to the intake of the sachet water are lactating babies (0–1 y) age group followed by the teenagers (12–17 y), Figure 2B. The two age groups, that is, (0–1 y) and (12–17 y) are in two important developmental stages (intensive bone growths) in the human body. Rapid bone growth requires a high level of calcium depositions; however, similar rates of ^226^Ra deposition on the bones also occur and consequently may in turn lead to bone and head-sinus cancers (10, 11, 45). Similar vulnerabilities for the two age groups were reported in the studies conducted by Asaduzzaman et al. (8) in 2016 on commercially bottled water in Bangladesh and by Khandaker (11) on bottled mineral water in Malaysia.

**Table 3:** Age–dependent annual effective dose (mSv/y) for the radionuclides.

	**Descriptive statistics**	**0–1 y**	**1–2 y**	**2–7 y**	**7–12 y**	**12–17 y**	**> 17 y**
^40^K	MAX	3.10	2.52	1.47	1.14	0.84	0.90
	MIN	0.25	0.21	0.12	0.09	0.07	0.07
	AVE1	1.03	0.83	0.49	0.38	0.28	0.30
	SDV	0.85	0.69	0.40	0.31	0.23	0.25
	GEOMEAN	0.73	0.59	0.35	0.27	0.20	0.21
	GSD	2.43	2.44	2.43	2.43	2.45	2.42
	SKEWNESS	1.07	1.06	1.07	1.06	1.07	1.06
	KURTOSIS	0.08	0.07	0.08	0.08	0.10	0.06
^226^Ra	Descriptive Statistics	0–1 y	1–2 y	2–7 y	7–12 y	12–17 y	> 17 y
	MAX	20.05	4.91	3.70	6.01	14.07	3.49
	MIN	1.80	0.44	0.33	0.54	1.26	0.31
	AVE2	8.09	1.98	1.49	2.42	5.68	1.41
	SDV	4.31	1.05	0.80	1.29	3.02	0.75
	GEOMEAN	6.96	1.71	1.29	2.09	4.89	1.21
	GSD	2.43	2.44	2.43	2.43	2.45	2.42
	SKEWNESS	0.85	0.85	0.85	0.85	0.85	0.85
	KURTOSIS	0.50	0.49	0.50	0.51	0.49	0.51
^228^Ra	Descriptive Statistics	0–1 y	1–2 y	2–7 y	7–12 y	12–17 y	> 17 y
	MAX	129.15	29.45	20.49	29.55	50.20	8.67
	MIN	14.63	3.33	2.32	3.35	5.68	0.98
	AVE3	71.15	16.22	11.29	16.28	27.65	4.78
	SDV	37.32	8.51	5.92	8.54	14.50	2.51
	GEOMEAN	59.28	13.51	9.41	13.56	23.04	3.98
	GSD	2.43	2.44	2.43	2.43	2.45	2.42
	SKEWNESS	0.01	0.01	0.01	0.01	0.01	0.01
	KURTOSIS	−1.54	−1.54	−1.54	−1.54	−1.54	−1.54

Table 4 shows the evaluated radiological risks (lifetime cancer risks), which are mortality and morbidity, due to the consumption of the radionuclides ^226^Ra and ^228^Ra in adults. The risks varied from 0.6 × 10^−3^ to 6.3 × 10^−3^ with an average value of (2.5 ± 1.3) × 10^−3^ and 0.8 × 10^−3^ to 8.7 × 10^−3^ with an average value of (3.5 ± 1.9) × 10^−3^, respectively for ^226^Ra. The risks changed from 2.0 × 10^−3^ to 17.6 × 10^−3^ with an average value of (9.7 ± 5.4) × 10^−3^ and 2.9 × 10^−3^ to 25.6 × 10^−3^ with an average value of (14.0 ± 7.3) × 10^−3^, respectively for ^228^Ra. Apart from the results from samples with concentration falling below the MDA, results indicate that all the values of the risks are higher than the permissible limit of 10^−3^ for safe use of the drinking water except for mortality risks obtained in Awa28 and Awa 31 samples and the morbidity risks obtained in Awa31 samples due to the intake of ^226^Ra. The average values are far higher than the values 2.1 × 10^−5^/3.0 × 10^−5^ (mortality/morbidity risk) for ^226^Ra and 5.4 × 10^−5^ /7.5 × 10^−5^ (mortality/morbidity risk) for ^228^Ra, as reported by Asaduzzaman (8) in bottled water sold in Bangladesh. It is also observed that the average values of mortality risk 7.47 × 10^−5^ due to ^226^Ra and 1.16 × 10^−4^ due to ^228^Ra in surface and groundwater surveyed by El-Gamal et al. (37) in Assiut, Governorate, Egypt are lower than the values obtained in the present study. Going by the results of this study as compared with the reference values, the intake of the investigated sachet water of all the surveyed brands by the members of the public may experience significant radiological hazards if no remedial action is taken by the producers.

**Table 4:** Radiological risks of radium isotope ^226^Ra and ^228^Ra in the sampled sachet water.

**Sample codes**	**Life–time cancer risks (ELCR x 10** ^**−3**^ **)**			
	**Mortality**	**Morbidity**	**Mortality**	**Morbidity**
	^**226**^ **Ra**		^**228**^ **Ra**	
Awa 1	4.8	6.7	13.5	19.4
Awa 2	1.7	2.4	15.8	22.7
Awa 3	4.9	6.8	13.1	18.8
Awa 4	1.2	1.7	14.9	21.5
Awa 5	6.3	8.7	15.0	21.6
Awa 6	ND	ND	14.8	21.3
Awa 7	4.0	5.6	17.6	25.4
Awa 8	3.2	4.4	16.3	23.5
Awa 9	1.9	2.7	9.4	13.5
Awa 10	3.2	4.5	6.5	9.3
Awa 11	2.3	3.3	17.0	24.5
Awa 12	1.2	1.6	14.7	21.1
Awa 13	2.5	3.5	8.4	12.1
Awa 14	3.8	5.4	8.1	11.7
Awa 15	3.6	5.1	15.3	22.1
Awa 16	2.0	2.8	12.5	18.0
Awa 17	ND	ND	15.1	21.7
Awa 18	2.7	3.8	4.7	6.8
Awa 19	1.9	2.6	ND	ND
Awa 20	3.1	4.3	7.4	10.7
Awa 21	ND	ND	2.5	3.6
Awa 22	1.3	1.8	ND	ND
Awa 23	1.1	1.6	2.7	3.9
Awa 24	ND	ND	4.4	6.3
Awa 25	2.0	2.8	8.6	12.4
Awa 26	3.5	4.9	ND	ND
Awa 27	1.2	1.6	2.7	3.9
Awa 28	0.8	1.1	ND	ND
Awa 29	2.4	3.4	8.4	12.1
Awa 30	ND	ND	2.0	2.9
Awa 31	0.6	0.8	5.4	7.7
Awa 32	ND	ND	4.7	6.8
Awa 33	3.2	4.4	6.5	9.3
Awa 34	1.3	1.9	ND	ND
Awa 35	1.5	2.1	3.2	4.6
AM ± SD	2.5 ± 1.3	3.5 ± 1.9	9.7 ± 5.1	14.0 ± 7.3

*NA, Not Applicable*.

## Conclusion and Recommendation

In this study, an evaluation of the radiological health risks of the most popular brands of sachet water in the Ondo state of Nigeria has been carried out. A total of 35 brands of sachet water samples were investigated for the content of ^40^K, ^226^R, and ^228^Ra. The concentrations of the radionuclides varied considerably from one brand to another with the corresponding average annual effective doses exceeding the recommended limit of WHO (57). Comparative analysis was carried out on the determined concentrations of the radionuclides in a few samples, which by chance appeared between this research and in a more than 10-year-old study. Despite the given recommendations in the previous study to employ suitable methods of reducing excessive radioactive contents from those brands, this study has observed that there is no statistically significant change in the concentrations of radionuclides in the same brand water samples. Both the mortality risks and the morbidity risks due to the presence of radium isotopes in the water samples were observed to have higher values than the limiting value (10^−3^) for safe use of drinking water except in a few samples. It is, therefore, recommended that the consumers of the products should avoid drinking these specific brands of water, if possible, or at least should reduce their consumption. All producers of the products should screen their water sources (drilled and dug wells or reservoirs) for loads of radionuclides/other contaminants, and take appropriate treatment/actions where ever necessary. There are many brands of sachet water in Nigeria from various manufacturers in different locations, and many other factories are being proposed for sachet water production to meet the demands of the public, therefore any newly proposed sites/aquifers for sachet water production should be screened by appropriate bodies before the commencement of operational activities. As a regulatory body for food and drug control in Nigeria, the National Agency for Food and Drug Administration and Control should ensure constant monitoring and quality control of the levels of exposure to radiation *via* ingestion of water and ensure that the given international standard regarding radioactive content of sachet drinking water is complied with. The data of this research will form an invaluable component of radiometric values of the growing database in Nigeria as well as the world for setting up guidelines and control policies for the use of sachet water.

## Data Availability Statement

The original contributions presented in the study are included in the article/supplementary material, further inquiries can be directed to the corresponding author.

## Author Contributions

KA: conceptualization of the study. MK and KA: writing of original draft. CO and SA: software validation. SA and MK: editing of the manuscript. KA and CO: sampling. KA, MK, and SA: data analysis. MK, CO, and SA: review of the manuscript. All authors contributed to the article and approved the submitted version.

## Conflict of Interest

The authors declare that the research was conducted in the absence of any commercial or financial relationships that could be construed as a potential conflict of interest.

## Publisher's Note

All claims expressed in this article are solely those of the authors and do not necessarily represent those of their affiliated organizations, or those of the publisher, the editors and the reviewers. Any product that may be evaluated in this article, or claim that may be made by its manufacturer, is not guaranteed or endorsed by the publisher.
